# Beyond the Camera Trap: A Systematic Review of Computing Technology Used to Monitor and Interact with (More) Varied Taxa in Zoos and Aquariums

**DOI:** 10.3390/ani15121721

**Published:** 2025-06-11

**Authors:** Lilliana Hassinger, Christena Nippert-Eng

**Affiliations:** Department of Informatics, Luddy School of Informatics, Computing, and Engineering, Indiana University, Bloomington, IN 47408, USA; cnippert@iu.edu

**Keywords:** zoos, aquariums, technology, cameras, animal–computer interaction, ACI, animal welfare, species, mammals, systematic literature review

## Abstract

Zoos and aquariums house a wide range of species, yet research remains heavily skewed toward mammals and camera-based technologies. This systematic literature review examines the use of computing technologies to monitor or interact with animals in zoos, aquariums, or wildlife parks, with a focus on taxonomic representation and technological diversity. A total of 125 studies published between 2014 and 2024 met the inclusion criteria, encompassing 151 recorded instances of technology use. Cameras and video systems were the most frequently used tools, appearing alone in 40% of studies and in combination with other technologies in an additional 18.4%, accounting for 48.3% of all technology instances. Most studies focused on only mammals (73.5%), and behavioral monitoring was the most common research aim (40.9%). These findings suggest an uneven distribution of research shaped more by convenience and familiarity than by welfare need, highlighting a critical opportunity to diversify both species focus and technological application. Increased investment in underrepresented species and underutilized tools will help ensure that research better reflects the full spectrum of animal needs and experiences.

## Simple Summary

Broad adoption of diverse technologies to monitor and interact with a wide range of species is essential for advancing zoo and aquarium animal welfare and conservation goals. However, a systematic literature review of 125 peer-reviewed articles published between 2014 and 2024 reveals that cameras were disproportionately represented, with 73 instances of camera or video monitoring across 125 studies. In 40% of studies, cameras were the only technology used, while an additional 18.4% combined cameras with other tools. Overall, camera-based technologies accounted for 48.3% of all technology use instances (*n* = 151). Most studies focused exclusively on mammals (73.5%). Clearly, technological and taxonomic diversity are not yet fully realized in this body of published research. Incorporating a broader range of tools to support less frequently studied taxa would help researchers better understand and meet the needs of all animals living in managed care. This review highlights how convenience, familiarity, and historical norms may influence which species and research questions receive attention, potentially limiting progress toward equitable welfare for all taxa.

## 1. Introduction

In zoos and aquariums (including wildlife parks, unless otherwise noted), technological advancements are increasingly leveraged to monitor and engage with animals in managed care, offering innovative approaches to behavioral research and environmental enrichment [1,2,3]. Scientific research in zoos and aquariums spans a broad range of aims, reflecting their evolving role as institutions of conservation, education, and animal care. As Rose et al. (2019) emphasize, husbandry and welfare remain central themes of zoo-based research, aligning with the growing recognition of animal welfare as a fifth core aim of modern zoos [4,5]. Beyond welfare, studies also address behavioral ecology, veterinary care, physiology, and nutrition, highlighting both applied and foundational science that advances knowledge across disciplines [4].

Traditional methods of animal monitoring rely heavily on direct behavioral observations, physiological measurements, and environmental assessments [6,7,8]. While informative, these methods present limitations such as observer bias, time constraints, and the inability to track animals continuously [9,10]. The integration of computing technology into animal welfare research in these settings presents a promising avenue for overcoming these challenges, offering objective, automated, and non-invasive tools for tracking animal behavior, physiological states, and responses to environmental stimuli [1,2,11,12]. Over the past decade, in fact, the field of animal–computer interaction (ACI) has emerged precisely to explore how technology can facilitate, enhance, and analyze interactions between animals, their environments, and their human caretakers [13,14]. Over the past decade, a growing suite of digital and sensor-based technologies have emerged in research and husbandry contexts. Camera-based monitoring, accelerometers, RFID, bioacoustics, and thermal sensors have demonstrated success in agricultural and wild environments, though they are still being fully integrated into zoo and aquarium operations [1,2,3,11,12,15,16,17,18]. Some technologies—such as touchscreens for primates or audio-based interfaces for birds—also serve dual roles in cognitive enrichment and behavioral data collection, aligning with the ACI framework’s emphasis on agency and engagement [19,20,21].

Unfortunately, evidence suggests this technological momentum has been unevenly distributed across taxa, with research across a variety of settings disproportionately focused on large mammals while leaving reptiles, birds, fish, and amphibians comparatively understudied [1,2,3,6,21,22]. Additionally, barriers such as infrastructure costs, species-specific constraints, and the need for staff training have delayed the broader implementation of these technologies [4,11,19]. While several studies have raised these concerns, few have systematically evaluated how these patterns play out across a wide body of published research. This review addresses that gap by analyzing trends in taxonomic focus, technological tools, and research aims across ten years of relevant peer-reviewed literature.

The ACI community explicitly embraces a non-speciesist approach, prioritizing the needs, agency, and perspectives of nonhuman animals as equal to those of humans. Mancini’s ACI manifesto calls for treating all species with respect, using technology to benefit the animals involved, and ensuring their participation is voluntary and non-coercive [13]. This ethical stance challenges traditional hierarchies in animal research by centering the animal’s perspective in both design and implementation [13]. Hirskyj-Douglas’s work amplifies and exemplifies this position, in part by developing interfaces that reflect species-specific affordances and enable animals to interact with technology on their own terms [14,23]. In practice, ACI theory informs technology design by prioritizing species-specific preferences, sensory affordances, and mechanisms of control that center animal agency. For instance, the JoyBranch system developed for a hyacinth macaw at the San Diego Zoo exemplifies this approach by mimicking natural enrichment objects and allowing the bird to trigger and terminate music via ergonomic interactions tailored to its beak and posture. This co-designed device empowered the macaw to control its auditory environment, with usage increasing over time, demonstrating intrinsic motivation and engagement without training or rewards [21]. Similarly, Coe and Hoy (2020) argue that computing technologies like RFID-controlled feeding systems or motion-triggered interfaces should not only automate care but expand animals’ opportunities for choice, control, and environmental interaction, aligning with the ethical goals of ACI [19]. In a different example, Hirskyj-Douglas and colleagues (2022) created a computer interface for white-faced sakis that allowed them to select from multiple audio or visual stimuli. The system captured how these monkeys interacted over time, providing insight into their preferences while highlighting the role of novelty and the need for long-term, voluntary engagement as a measure of design success [23]. Collectively, these examples show how ACI not only shapes ethical intentions but directly informs the engineering and evaluation of technologies that are meaningful to the animals themselves.

When technological tools and research efforts disproportionately favor certain taxa, this not only limits welfare outcomes, but also risks undermining the public trust that zoos rely on to fulfill their conservation and education missions. As Veasey (2022) argues, zoos and aquariums have a moral obligation to ensure the welfare of all animals in their care—an obligation that must not be compromised by operational convenience or historical bias. Public approval and institutional credibility depend on demonstrating ethical consistency and scientific rigor across species [24].

Prior work has highlighted broad patterns in how technology is used in zoo and aquarium research, but a comprehensive synthesis is still needed to evaluate the depth and consistency of these trends. This review therefore focuses on three interrelated questions: (1) What computing technologies are currently used in zoos and aquariums to monitor and/or interact with animals? (2) Which animal taxa are most frequently studied using these tools? (3) What are the primary purposes or research aims associated with the use of such technology? While the first two questions provide necessary background, the third question is the most central for understanding how technology contributes to animal care and welfare outcomes.

To answer these questions, we conducted a systematic literature review of published research over the last ten years appearing in the top five zoo and aquarium animal behavior and welfare journals, examining the broader implementation of computing technology in animal care and research. Our findings reveal that mammalian species continue to dominate researchers’ attention, while tools beyond standard camera-based systems remain underutilized. This unevenness in both taxonomic and technological focus suggests that research priorities may still be driven by familiarity, accessibility, and legacy practices rather than by the full range of welfare needs across species. These patterns raise important questions about whether all animals in managed care are receiving equal consideration in research and highlight the need for more intentional inclusion of overlooked species and methods.

Our review follows a systematic literature review methodology, adhering to predefined inclusion and exclusion criteria for empirical, peer-reviewed studies conducted within zoos, aquariums, and wildlife parks. Our goal is to provide a comprehensive overview of the role of technology in these research settings. We emphasize technology’s potential to enhance current assessment methodologies and offer recommendations for future research and implementation strategies. In doing so, we position this work within the broader field of ACI, illustrating how technological innovations can be designed and adapted to improve the lives of even more animals in managed care. Addressing the persistent taxonomic biases in this research is essential for advancing equitable welfare outcomes, ensuring that technological tools are applied across the diversity of species housed in zoos and aquariums.

## 2. Background

Understanding the rationale behind the use of technology in zoos and aquariums requires exploring both the operational needs and welfare goals of these institutions. Monitoring technologies help ensure animal welfare, support conservation research, and facilitate husbandry practices by offering non-invasive, continuous data collection that can enhance animal care beyond the limitations of human observation [2,6,7,8,12,25,26]. These technologies provide deeper insights into animal health, behavior, and environmental conditions, enabling zoos to emphasize the importance of both physical and psychological wellbeing [1,2,27]. Many of these applications fall within the scope of animal–computer interaction (ACI), a field that promotes species-centered design, focusing on animal agency, voluntary engagement, and ethical interaction with technology [13,14].

### 2.1. Current Applications of Technology

Today, zoos and aquariums use a wide variety of technologies for both monitoring and interaction purposes. Wearable sensors such as GPS collars, RFID tags, accelerometers, and multi-sensor biologgers can track movement, enclosure use, energy expenditure, and behavioral changes [26,28]. Video-based tools such as CCTV systems, camera traps, and motion-triggered trail cameras are used widely to assess animal behavior in 24/7, non-invasive ways [2,25]. Advanced examples include AI-based facial and behavioral recognition systems deployed with individual animals, such as the orangutans at the Toronto Zoo, which are capable of identifying individuals and analyzing real-time pose and movement data [12,29].

Technologies also support cognitive enrichment, another core aspect of welfare [30]. Touchscreen systems for primates or sonic enrichment devices for birds allow animals to voluntarily engage with their environments [6,20,31,32]. These interactive technologies, which often incorporate embedded sensors to monitor engagement levels, exemplify ACI’s emphasis on designing systems that foster agency and control [19,26,28]. Sonic enrichment systems, like those designed for birds and elephants, not only provide sensory stimulation but enable animals to influence their environments, offering metrics for assessing engagement and welfare outcomes [21,33,34].

In parallel with these interaction-based tools, digital systems have also transformed behavioral observation—a core component of animal care. Physical ethnography, defined by Nippert-Eng as the intensive observation of individuals in their lived environments, has long been a foundation of welfare research in zoos [35]. Tools like ZooMonitor, developed by Lincoln Park Zoo, formalize and scale this observational work by enabling standardized behavioral tracking, space-use visualization, and cross-observer reliability scoring [36]. These systems bring scientific rigor and usability to behavioral monitoring, allowing keepers, researchers, and institutions to generate consistent baselines and detect subtle changes over time [37]. As one of the most widely adopted software platforms in the field, ZooMonitor exemplifies how longstanding ethnographic practices are now supported by accessible, welfare-oriented technologies.

Together, these technologies reflect a shift in zoo and aquarium research from observational to data-rich and responsive systems that can adapt to individual animal needs. Their increasing integration into daily husbandry not only enhances welfare monitoring but also reshapes the role of technology from a passive tool to an active partner in animal care. This progression underscores the growing potential for computing systems to support individualized, long-term welfare strategies that align with institutional goals for evidence-based management and ethical engagement.

### 2.2. Research Trends and Gaps

Although the literature strongly supports the effectiveness of these technologies, it also identifies several persistent gaps. Prior systematic literature reviews, including those by Diana et al. and Binding et al., have drawn attention to significant taxonomic bias in zoo and aquarium research [1,22]. These studies found that most research and technology use focus on large mammals—particularly primates and carnivores—while reptiles, amphibians, and aquatic species remain underrepresented. This imbalance limits welfare advancements for taxa with distinct behavioral and physiological needs. While these reviews provided essential insights into welfare-related research trends, they either limited their inclusion to studies explicitly referencing welfare terms (Diana et al.) or assessed a broader set of welfare literature without focusing specifically on computing technologies (Binding et al.) [1,22]. In contrast, the present review systematically examines how computing technology is used across taxa and research purposes, offering a more targeted and expansive view of technological implementation. Many tools—such as biologgers, infrared sensors, and audio monitoring—remain underutilized despite their cross-taxa potential.

Barriers to widespread technology adoption include financial cost, infrastructure (e.g., Wi-Fi availability), and data privacy concerns, especially for sound and video data collected in visitor-accessible enclosures [21,25]. Additionally, training animals to wear or interact with monitoring devices presents challenges; however, studies emphasizing voluntary engagement—such as crate-training for biologger attachment or interacting in sonic environments—demonstrate how these challenges can be mitigated while aligning with welfare goals [28,33]. The field of ACI offers further reasoning for broadening the scope of technology applications, as it emphasizes species-specific design that can support cognitive and sensory engagement for all animals [13,14]. Many of the aforementioned technologies and approaches, especially those focused on interaction and enrichment, fall under the ACI bucket. These technologies are designed not only for monitoring but also map more closely onto welfare frameworks by supporting choice, control, and engagement [19]. These examples suggest that integrating ACI principles into technology use offers a promising path forward, particularly for underutilized or emerging tools.

## 3. Materials and Methods

### 3.1. Data Collection

**Figure 1:**
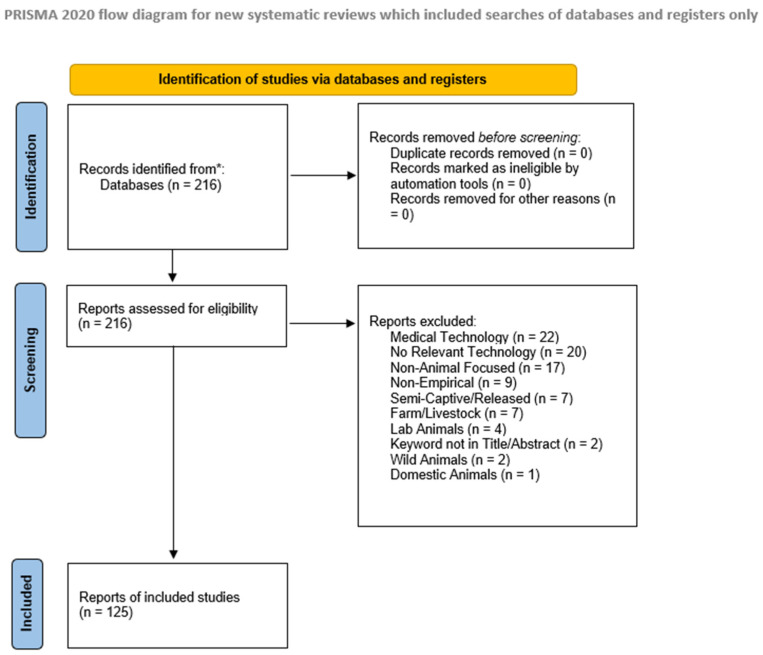
PRISMA 2020 flow diagram illustrating the study selection process for the systematic review on computing technology use in zoo and aquarium research. Note for *: Records are identified from *Animals*, *Zoo Biology*, *Journal of Applied Animal Welfare Science* (JAAWS), *Applied Animal Behaviour Science* (AABS), and *Journal of Zoo* and *Aquarium Research* (JZAR).

We conducted a systematic review of published research journal articles to investigate the use of technology in zoos and aquariums. This review followed the PRISMA 2020 guidelines for systematic reviews [38] (see Figure 1). A review protocol was not registered, however, as this study focused on research trends in technology use and taxonomic representation rather than health outcomes or interventions.

The five journals selected for our systematic review—*Animals*, *Zoo Biology*, *Journal of Applied Animal Welfare Science* (JAAWS), *Applied Animal Behaviour Science* (AABS), and *Journal of Zoo* and *Aquarium Research* (JZAR)—were chosen based on their demonstrated relevance to zoo and aquarium animal welfare and the use of technology in managed care settings. In Diana et al. (2021), *Zoo Biology* and *Animals* were reported as the most frequently used journals for publishing studies on the use of technology in zoos, comprising 31.5% and 26.1% of the reviewed articles, respectively, while JAAWS, AABS, and JZAR were each represented among the selected studies (5.3% each), affirming their value to this niche field [1]. Alligood and Leighty (2015) similarly found that *Zoo Biology*, JAAWS, and AABS were the top three journals publishing zoo enrichment evaluation research between 2002 and 2014, accounting for most of their reviewed studies (32, 19, and 16 articles, respectively) [39]. As enrichment is widely recognized as critical to animal welfare, supporting species-typical behaviors and psychological wellbeing, we felt that using this journal set was justified based on its historical strength in publishing behavioral and welfare-focused research, especially related to enrichment evaluation [40,41]. This emphasis on enrichment and welfare informed the selection of studies in this systematic review, as technologies that facilitate enrichment or behavioral monitoring are central to improving welfare outcomes. In a broader review by Binding et al. (2020), which analyzed over 4000 articles across seven journals, JZAR had the highest percentage of zoo- and aquarium-based welfare articles (26.9%), followed by *Zoo Biology* (17.8%) and JAAWS (16.6%) [22]. While *Animals* had a lower proportion (0.4%), it is a prominent outlet for open-access zoo research, making it relevant for us, too. Together, these journals provide a solid foundation for a systematic review focused on technology in zoos and aquariums, offering historical breadth and current relevance across behavior, enrichment, and welfare research domains.

Individual published papers from these journals were included our study if they met the following criteria: (1) the papers involved animals housed in zoos, aquariums, wildlife parks, or sanctuaries where the animals are not intended for release; (2) the authors studied animals either solely in these facilities or in combination with wild counterparts; (3) the authors employed computing technology to monitor or interact with animals—such as camera traps, biosensors, or interactive enrichment devices—with Infrared Thermography considered an acceptable exception to general exclusions on medical technology; and (4) the papers were published in the last ten years in the five previously mentioned peer-reviewed journals.

Our exclusion criteria removed studies that (1) involved animals that were released post-rehabilitation or part of head-start programs; (2) used semi-captive or wild-only populations; (3) involved domestic companion animals, farm animals, or laboratory animals; (4) were not original, empirical studies such as books, literature reviews, opinion pieces, or meta-analyses; (5) focused on certain medical technologies such as X-rays or MRIs; (6) used fecal or blood measurements (unless these were paired with computing technologies); or (7) examined software used solely for breeding or population management planning purposes. This approach ensured that only studies aligning with the goals of evaluating computing technology used to actively monitor or engage with animals in managed care were included.

The studies included in this review were published across five journals between 2014 and 2024, with proportional analysis revealing important patterns when accounting for the total number of papers published in each journal annually. While *Animals* and *Zoo Biology* contributed the largest absolute number of papers, JZAR consistently published a higher proportion of relevant studies relative to its overall output. For example, JZAR exceeded 14% of its total publications in both 2017 and 2024, highlighting its focus on zoo and aquarium research. In contrast, *Zoo Biology* fluctuated between 1% and 13%, despite being a larger journal by publication volume. *Animals*, which has seen exponential growth in its publication volume over the past decade, demonstrated low proportional representation of studies meeting this review’s criteria, consistently below 1% each year. This suggests that while *Animals* publishes widely across animal science disciplines, its coverage of computing technology in zoos and aquariums remains limited. Additionally, while *Applied Animal Behaviour Science* (AABS) was included in the search scope, no studies from AABS met the inclusion criteria. This may reflect the limited indexing of AABS on PubMed, the primary database used for this review. Compared to its total publication volume, only a small fraction of AABS articles appear in PubMed, which may have restricted the capture of relevant studies from that journal (see Table 1).

### 3.2. Literature Search Strategy

To identify relevant studies, a comprehensive search was conducted using PubMed and the *Journal of Zoo* and *Aquarium Research* (JZAR) website. For PubMed, the search focused on four key journals: *Animals*, *Zoo Biology*, *Journal of Applied Animal Welfare Science* (JAAWS), and *Applied Animal Behaviour Science*. The search terms included a combination of keywords related to zoos, aquariums, and captive settings, as well as specific computing technologies used in animal monitoring or interaction.

The PubMed query was constructed as follows:

- Journal Inclusion: Articles were filtered to include publications from the selected four journals.
  ○ (“Animals: an open access journal from MDPI”[Journal] OR “Zoo Biology”[Journal] OR “Journal of applied animal welfare science: JAAWS”[Journal] OR “Applied animal behaviour science”[Journal])
- Zoo and Aquarium Context: Articles had to reference terms indicating relevance to zoos, aquariums, or similar captive settings.
  ○ (“zoo”[Title/Abstract] OR “zoos”[Title/Abstract] OR “zoological*”[Title/Abstract] OR “animal park*”[Title/Abstract] OR “aquarium*”[Title/Abstract] OR “marine park*”[Title/Abstract] OR “captive*”[Title/Abstract] OR “captivit*”[Title/Abstract])
- Technological Applications: The search included terms for computing technologies applied in monitoring or interacting with animals.
  ○ (“camera*”[Title/Abstract] OR “video*”[Title/Abstract] OR “biosensor*”[Title/Abstract] OR “GPS”[Title/Abstract] OR “tracking device*”[Title/Abstract] OR “RFID”[Title/Abstract] OR “chip*”[Title/Abstract] OR “motion detector*”[Title/Abstract] OR “thermal”[Title/Abstract] OR “imaging*”[Title/Abstract] OR “microphone*”[Title/Abstract] OR “sound sensor*”[Title/Abstract] OR “accelerometer*”[Title/Abstract] OR “remote sensing”[Title/Abstract] OR “drone*”[Title/Abstract] OR “computer vision”[Title/Abstract] OR “playback*”[Title/Abstract] OR “microchip*”[Title/Abstract] OR “telemetry”[Title/Abstract] OR “ultrasonic”[Title/Abstract] OR “automated”[Title/Abstract] OR “automation”[Title/Abstract] OR “feeding system*”[Title/Abstract] OR “touchscreen*”[Title/Abstract] OR “interface*”[Title/Abstract] OR “virtual”[Title/Abstract] OR “augmented”[Title/Abstract] OR “reality device*”[Title/Abstract] OR “haptic”[Title/Abstract] OR “feedback system*”[Title/Abstract] OR “robot*”[Title/Abstract] OR “projection*”[Title/Abstract] OR “music”[Title/Abstract] OR “simulated”[Title/Abstract] OR “digital”[Title/Abstract] OR “artificial intelligence”[Title/Abstract] OR “machine learning”[Title/Abstract] OR “image recognition”[Title/Abstract] OR “software*”[Title/Abstract] OR “video analysis”[Title/Abstract] OR “big data”[Title/Abstract] OR “3D”[Title/Abstract] OR “deep learning”[Title/Abstract] OR “smart collar*”[Title/Abstract] OR “wearable*”[Title/Abstract] OR “health device*”[Title/Abstract] OR “infrared”[Title/Abstract] OR “bio-logg*”[Title/Abstract])
- Date Range: The search was limited to studies published between 1 January 2014, and 31 October 2024.
  ○ (“1 January 2014”[Date-Publication]: “31 October 2024”[Date-Publication])

For the *Journal of Zoo* and *Aquarium Research* (JZAR), which is not indexed in PubMed, an adapted query was implemented using the journal’s search feature to ensure alignment with the inclusion criteria. Web scraping tools were utilized to systematically collect and export the search results for screening and further analysis. This approach allowed for the inclusion of studies from JZAR, ensuring a comprehensive dataset.

The literature search was conducted in November of 2024. All screening and data extraction were conducted by a single reviewer. Initially, 216 papers were exported and systematically screened based on the inclusion and exclusion criteria. Titles and abstracts were assessed using Excel-based keyword formulas, followed by manual verification of abstracts for accuracy. Exclusions were documented with specific reasons. If a paper’s title or abstract was missing any of the required keywords or concepts, it was flagged for manual review. Additionally, all abstracts were manually skimmed for further verification of relevance. The risk of bias within individual studies was not assessed, as this review focused on describing research trends in taxonomic and technological representation, rather than evaluating study outcomes. Following this process, 125 papers met the inclusion criteria and were selected for analysis; a total of 91 papers were excluded from further review. 

### 3.3. Data Extraction

For each selected study, the following information was extracted and categorized:

#### 3.3.1. Animal Information

The family, order, and taxonomic class of each species studied (e.g., Mammal, Avian, Reptile, Amphibian, Fish, or Insect) were manually recorded. For analyzing taxonomic representation, each study was counted only once per relevant taxonomic category, regardless of how many species it included. For example, if a study included three different primate species, it was counted once under “Primates” and once under “Mammals”. Similarly, a study examining five different bird species was counted once under “Avian”. In cases where a study included multiple classes—such as three mammals and two reptiles—it received one count for “Mammals” and one for “Reptiles”. This approach ensured that studies involving multiple species did not disproportionately skew the representation of any taxonomic group in the results.

#### 3.3.2. Technology Used

Each study was categorized based on the technology or technologies used, using the following classifications and matched keywords.

- Cameras and/or Video Monitoring: Includes video and camera-based systems such as CCTV footage, video recordings, and camera traps.
- Infrared Technology: Covers infrared and thermal imaging tools such as thermal imaging systems and infrared cameras.
- Touchscreen Systems: Includes touchscreen or tablet-based systems like interactive enrichment devices.
- Audio Playback/Recording: Encompasses auditory systems such as audio playback tools and sound recording devices.
- Wearables, Biologgers, and RFID: Combines technologies like GPS collars, wearable biologgers, and RFID-based tagging.
- Other: Captures uncategorized technologies, including automated systems, 3D imaging, artificial intelligence, and data loggers.

#### 3.3.3. Study Purposes

The purpose of each study was also categorized into one or more of the following categories based on keywords found in the abstract and title.

1. Behavior, which focused on animal behavior, activity patterns, enrichment, welfare, and stereotypies.
2. Environment, which examined the impact of environmental variables like temperature, habitat, noise, and visitor presence.
3. Reproduction and breeding, which studied breeding, maternal care, offspring development, and parturition (i.e., giving birth).
4. Technology testing and development, which included studies that evaluated or validated technologies, such as prototypes and calibration tools.
5. Health and physiology, which focused on stress, growth, thermoregulation, and body condition.
6. Conservation and management, which addressed conservation efforts, species management, population sustainability, and captive breeding programs.
7. Other: Studies that did not match the predefined keywords or categories.

### 3.4. Categorization Process

The categorization process involved matching each study’s abstract and title with the above words for both technologies and purposes. We used Excel formulas to automatically flag relevant studies based on keyword matches and manually input data for complex or unclear cases. For further analysis, Excel formulas such as COUNTIFS and pivot tables and charts were utilized to explore various aspects of the data and visualize trends effectively.

## 4. Results

### 4.1. Technology Breakdown

**Figure 2:**
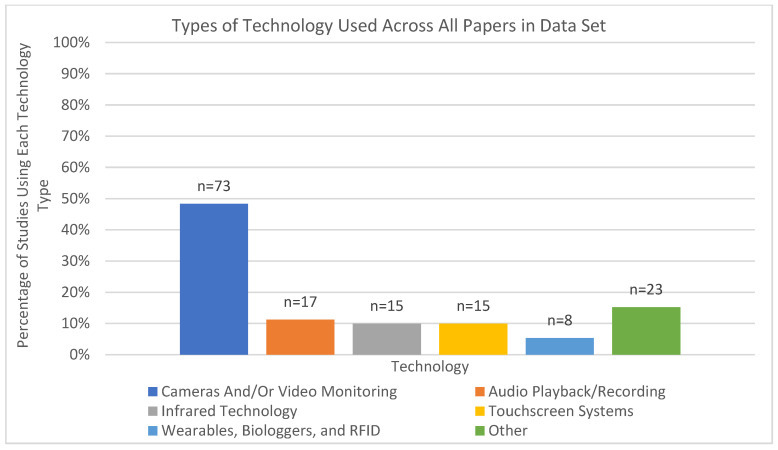
Non-exclusive types of technology used across all papers in the dataset.

A total of 151 unique counts of the kind of technology used in each study were recorded in the dataset. In this dataset, the term *cameras* is used broadly to indicate both still image and video-based monitoring systems. Cameras and/or video monitoring were the most frequently utilized technologies, accounting for 48.3% of studies (*n* = 73). This was followed by infrared technology and touchscreen systems, each representing 9.9% of studies (*n* = 15). Audio playback/recording technologies were employed in 11.3% of studies (*n* = 17), while wearables, biologgers, and RFID were used in 5.3% of studies (*n* = 8). The remaining 15.2% of technologies used in the studies (*n* = 23) fell into the “Other” category, which included less common technologies such as automated systems, 3D imaging, artificial intelligence, and data loggers. This distribution highlights a predominant reliance on visual monitoring technologies, with other categories including those focused on other sensory data (e.g., sound) representing more specialized or experimental approaches (see Figure 2).

**Figure 3:**
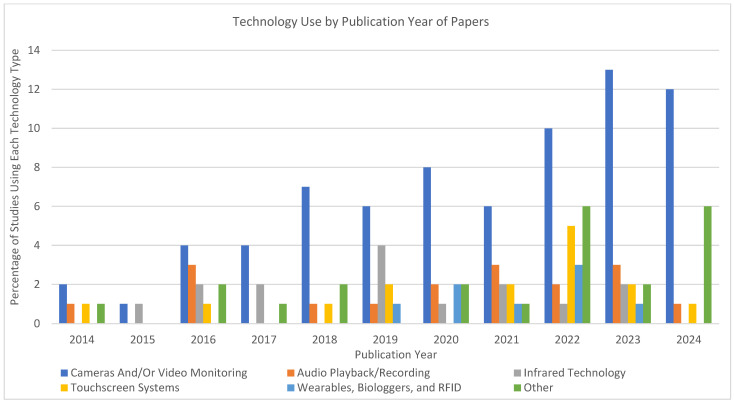
Non-exclusive technological distribution by year of publication across studies included in this systematic review (2014–2024).

A notable trend in recent years is the increased use of AI, deep learning, and automated behavioral tracking in zoo and aquarium research [12]. Of the studies in our dataset in the “Other” category, nearly one third of the studies employed advanced automated video analysis tools, and all were published in 2022 or later, highlighting a recent surge in AI-driven monitoring approaches [16,42,43,44,45,46,47]. These studies all focused on mammals, where deep learning models and machine learning-assisted behavioral tracking were applied [16,29,42,43,44,45,46,47] (see Figure 3). While not included in our dataset, recent research using 3D photogrammetry and automated model generation to quantify usable space in complex primate habitats further illustrates the expanding role of computer vision and automation in zoo-based welfare applications [48]. These AI and automation technologies were used to process large datasets, identify specific behaviors, and reduce manual coding effort in species noted for complex behavioral repertoires. This shift suggests that AI and automation are emerging tools in welfare research, although their adoption remains largely confined to mammalian subjects.

Figure 3 illustrates the technology used by publication year, emphasizing the persistent reliance on cameras and video monitoring. While other technologies, such as infrared sensors, bioacoustics, touchscreen systems, and wearables appear sporadically, their use remains limited and inconsistent across years.

### 4.2. Taxonomic Breakdown

**Figure 4:**
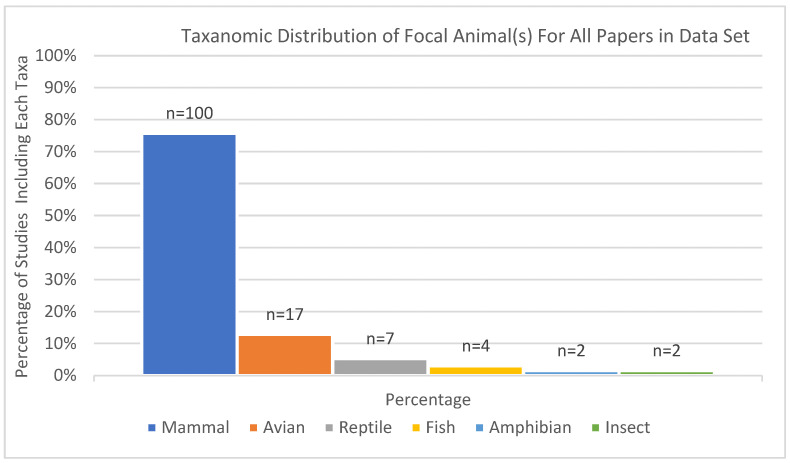
Taxonomic distribution of non-exclusive focal animal(s) across all studies included in the systematic review.

Mammals constituted the majority of the unique taxonomic classes represented in the dataset (*n* = 132), accounting for 75.8% of studies (*n* = 100) of the total classifications. Birds (avian taxa) comprised 12.9% of studies (*n* = 17), while reptiles represented 5.3% of studies (*n* = 7). Fish made up 3.0% of studies (*n* = 4), while amphibians and insects each comprised 1.5% of studies (*n* = 2), indicating minimal representation of these groups (see Figure 4). This distribution highlights the significant predominance of mammalian taxa in the dataset, with other taxonomic classes making up much smaller proportions.

**Figure 5:**
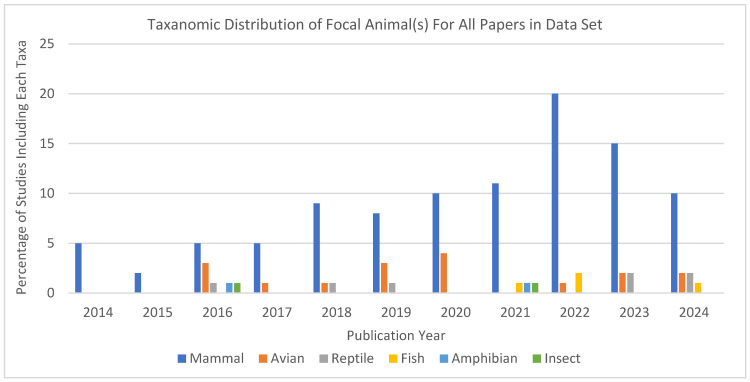
Non-exclusive count of taxonomic group by year of journal publication across studies included in the systematic review (2014–2024).

Figure 5 shows the representation of taxonomic groups in each year’s worth of published studies. Mammals consistently dominated, with peak representation in studies published in 2022. Avian taxa enjoyed their highest representation in 2020. Most interesting, perhaps, is that non-mammalian taxa were entirely absent from published studies during other years, most notably in 2014 and 2015, when only papers about mammals met our inclusion criteria.

### 4.3. The Focus on Vision

Cameras and video monitoring were this review’s most frequently used technology, appearing in 48.3% of studies (*n* = 73). Among these, 50 studies (33.1%) relied solely on cameras without incorporating additional monitoring tools. A subset of 23 studies (15.2%) combined cameras with additional technologies such as infrared imaging (*n* = 10), audio playback or recording (*n* = 5), or other monitoring tools (*n* = 7). Only one study used wearables, biologgers, and RFID with cameras. The “other” category included advanced camera technologies like motion sensor cameras, stereo–video imaging, automated video analysis, and deep learning models.

The taxonomic distribution of camera-based studies remained skewed toward mammals, particularly primates, elephants, and bears. An interesting outlier was the paper “The Various Ways in Which Birds Blink”, which used cameras to obtain video of species from every order of bird (43 orders) and 15 out of 24 species of crocodile [49].

### 4.4. Multimodal Technology Use

Nearly all multimodal studies identified in this review paired cameras or video systems with an additional technology, illustrating both the dominance of visual monitoring and the potential of integrating complementary data streams [29,42,43,46,50,51,52,53,54,55,56,57,58,59,60,61,62,63,64,65,66,67,68,69,70,71]. Three examples from this review demonstrate how multimodal technologies can enhance research outcomes by capturing behavioral and physiological dimensions that single technologies alone could not achieve. In a study of Asian elephants, Beeck et al. (2022) used acoustic cameras, rather than traditional video, to visualize and analyze the vocal pathways of elephant rumbles, revealing velopharyngeal coupling–simultaneous sound emission through the mouth and trunk [52]. This approach allowed for detailed spatial localization of sound sources, a layer of information that is not possible with a standard camera or audio recordings alone, underscoring the value of multimodal systems for studying vocal complexity [52].

Similarly, in a study on tamanduas (Tamandua tetradactyla), Pavese et al. (2022) combined tri-axial accelerometers with video validation to assess how husbandry conditions influenced activity patterns [53]. The accelerometers provided continuous data on animal activity levels, while video footage enabled the validation and calibration of these data streams [53]. This multimodal pairing allowed for precise activity monitoring in response to environmental changes, with the accelerometer providing movement data and the camera footage offering behavioral context and validation [53].

A third example involved monitoring captive Fiordland penguins and collared peccaries during exposure to live musical performances [54]. Cameras were combined with sound loggers to evaluate how the concert influenced behavior [54]. This pairing captured both environmental stimuli (music levels) and animal responses (behavioral changes) [54]. However, the study noted that the birds were often out of view of the cameras, leaving their location and behavior unknown during portions of the observations [54]. Incorporating RFID tracking or wearable technologies could address this gap by providing continuous spatial data, ensuring that all individuals are monitored even when they are not visible on camera.

Despite these successes, nearly all multimodal technology studies included in this review focused on mammals [29,42,43,46,52,53,55,56,57,58,59,60,61,62,64,65,66,68,69,70,71]. A few studies focused on or included avians, and only one each included fish and reptiles [50,51,54,63,72]. No similar multimodal designs were applied to amphibians or insects in this review; however, as there were only two studies each that included amphibians and insects in this review, that is not surprising [72,73,74]. Most multimodal studies also relied heavily on cameras or video monitoring as the foundational technology, reinforcing the visual-centric bias in zoo and aquarium research.

### 4.5. Purpose of Studies

Studies could be conducted for multiple purposes. The 125 studies we analyzed included 276 separate study purposes or reported reasons for using the technologies. Behavioral studies were the most common group of papers, accounting for 40.9% (*n* = 113) of the stated study purposes, underscoring the importance of understanding animal behavior in zoo and aquarium settings. Understanding environmental factors in these managed care settings was the second most frequent focus of our dataset, at 19.2% (*n* = 53). Conservation and management purposes were listed in 16.7% of studies (*n* = 46), health and physiology accounted for 9.8% of studies (*n* = 27), and reproductive and breeding interests made up 9.4% of studies (*n* = 26). Technology testing and development was less common, at 2.5% of studies (*n* = 7), while the “Other” category represented 1.4% of studies (*n* = 4). These findings reflect the diversity and multiple overlapping purposes of the research included in our dataset, despite the dominance of behavioral research.

For each broad category of study purpose—e.g., “behavior”, “welfare”, “environmental”—we also looked at 312 purpose-related terms that were included in the titles and abstracts. Because studies could serve multiple purposes, a single study could be associated with more than one term. We then grouped related terms together under their broader thematic categories to identify trends. For example, the category “behavior” included studies that used that word explicitly, as well as those mentioning “behavioral” or “behavioral monitoring”. Similarly, the “welfare” category encompassed studies referring to “welfare,” “wellbeing,” or “well-being”.

Not surprisingly, “behavior” was the most frequently coded term, associated with 20.8% of studies (*n* = 65). “Welfare” and its related terms collectively accounted for 18.6% of studies (*n* = 58), highlighting its prominence in zoo and aquarium research. Other frequent terms included “activity” and related phrases such as “activity patterns” and “activity budget*” (17.0% of studies, *n* = 53), and “enrichment” (9.0% of studies, *n* = 28). Social-related terms such as “social” and “affiliation” appeared in 8.0% of studies (*n* = 25), while “responses” were noted in 6.4% of studies (*n* = 20). Additional terms like “control” (5.4% of studies, *n* = 17), “nocturnal” (5.1% of studies, *n* = 16), “preference*” (4.5% of studies, *n* = 14), and “choice” (3.8% of studies, *n* = 12) helped to further characterize the behavioral focus of the research.

## 5. Discussion

This discussion explores key questions that emerged from the results: Why do visual technologies dominate? How does this affect welfare outcomes across taxa? What are the consequences of a persistent focus on behavioral indicators? And how can future work address these limitations? These questions are established in persistent patterns identified in the published literature, which highlight critical constraints in how computing technology is applied in zoo and aquarium settings. Most studies continue to focus on mammals, particularly charismatic megafauna such as elephants, primates, and large carnivores [6,75]. Although several reviews have emphasized the importance of expanding research on non-mammalian taxa [6,20], an examination of the published literature suggests that little progress has been made in expanding research interests since the most recent assessment in 2020. In addition to this taxonomic skew, we found that camera-based systems dominate the technological landscape, with limited integration of multimodal tools capable of capturing physiological or internal welfare indicators. Study purposes were also concentrated around behavioral monitoring, with relatively fewer studies focusing on health, reproduction, or environmental factors. Together, these patterns reveal a research field still constrained by limited taxonomic, technological, and topical diversity.

### 5.1. Comparison to Previous Research

This review was motivated by prior work identifying key gaps in zoo and aquarium research, especially regarding species representation and the use of technology to support animal welfare. Binding et al. (2020) surveyed the broader field of zoo welfare studies and found that 75% focused on mammals, with great apes comprising a substantial portion of the research effort [22]. This pattern persists in the current review, where mammals accounted for over 75% of the taxa studied. The continued dominance of mammalian subjects highlights an ongoing taxonomic bias that limits the generalizability of findings across the full range of species housed in zoos and aquariums, particularly birds, reptiles, amphibians, and fish [22].

Diana et al. (2021) conducted a systematic review examining the use of technology to assess welfare in zoo animals, focusing specifically on automated monitoring tools and emphasizing real-time, continuous systems [1]. Their review included only 19 publications, all of which were selected based on the explicit use of welfare-related terms (e.g., “welfare,” “wellbeing”) [1]. The majority of studies (89.5%) focused on mammals—particularly elephants and primates—and the most frequently used technologies were cameras (52.6%) and wearable sensors (31.6%) [1]. Notably, they found that the implementation of real-time algorithmic monitoring systems remained limited, with only two studies meeting that threshold [1].

In contrast to Diana et al. (2021), our review used broader search criteria [1]. By omitting welfare terms from the search strategy, we were able to include studies that used computing technologies in contexts such as behavioral research, enrichment, and physiological monitoring, even if welfare was not stated as a primary outcome. This approach aligns with the understanding that welfare can be indirectly assessed or supported through research on behavior, environmental interaction, and internal state monitoring. Additionally, the technological scope of this review was broader than previous systematic reviews. Diana et al. (2021) focused primarily on sensor-based technologies, including cameras, microphones, and wearable sensors [1]. In contrast, this review incorporated a wider range of computing technologies into the search parameters. This expanded approach captures emerging fields like animal–computer interaction (ACI) and reflects the growing integration of computational tools in zoo and aquarium research. This methodological distinction likely contributed to the larger dataset analyzed in this review and the greater technological diversity represented across studies.

### 5.2. Dominance of Visual Technology in Welfare Research

One of the central questions raised by this review is why visual technologies—especially cameras—remain so dominant in zoo and aquarium research. A notable trend in this study was the dominance of cameras as the primary technology used for monitoring welfare. Nearly half of the studies examined relied solely on cameras, while over half incorporated them alongside additional technologies. While cameras provide valuable behavioral data, they have limitations in assessing specific aspects of welfare, particularly physiological indicators. For example, cameras alone cannot assess internal states such as stress levels, thermoregulation, or cardiovascular function. One likely reason for their dominance is their widespread availability; many zoos and aquariums already use camera systems for security or husbandry purposes, allowing researchers to repurpose existing infrastructure or archival footage. Cameras also align with a human-centered preference for visual information, which may not reflect the most relevant or reliable signals of welfare for nonhuman animals. This visual bias becomes particularly problematic when it shapes which technologies are adopted to study other species, often favoring what is accessible and intuitive for us over what is meaningful or measurable for the species being studied.

Using alternative and complementary technologies, such as infrared thermography, RFID tracking, and bioacoustics, could provide a more holistic view of welfare status. Infrared thermography enables non-invasive monitoring of body temperature, offering insights into thermoregulation and stress responses [76,77]. RFID systems can track fine-scale movement and social interactions, while bioacoustic monitoring captures vocal indicators of emotional state and social dynamics [26,78]. These tools can address gaps left by purely visual assessments, creating a fuller picture of animal welfare that encompasses both observable behavior and internal physiological states. Infrared cameras, for example, were essential for capturing nocturnal breeding behaviors in tawny frogmouths (Podargus strigoides), an understudied species, as traditional observation methods would have disrupted their activity or failed to detect key behaviors in low-light conditions [50]. Similarly, infrared video systems enabled overnight behavioral monitoring of aardvarks—a relatively underrepresented mammal—in an enrichment study assessing affiliative and agonistic behaviors, paired with glucocorticoid metabolite analysis to evaluate welfare [79]. Without infrared technology, continuous, non-invasive monitoring of these nocturnal behaviors in species like tawny frogmouths and aardvarks would not have been feasible.

While ACI promotes a non-speciesist animal-centered approach to interactive technology, its application in zoos remains limited, especially for non-primate or non-mammalian species. One of the few empirical ACI studies involving a zoo-housed non-primate is Morrison et al.’s “Platypus Surfing: In Search of the Perfect Wave”, in which a solo platypus at Melbourne Zoo used a sensor-triggered wave enrichment system—an example of species-specific, non-visual interactive design [80]. However, such implementations remain rare; most ACI efforts involving non-mammals are conceptual or exploratory rather than applied. Going forward, researchers and designers have the opportunity to rebalance this visual dominance by embracing tools that reflect animals’ own sensory capacities, enabling more meaningful assessments of welfare across a wider range of species.

### 5.3. Charismatic Species Bias and Welfare Implications

This technological pattern parallels another key issue: the persistent focus on charismatic mammalian species, which continues to shape how welfare is studied and prioritized. The over-representation of mammals in zoo and aquarium research reflects broader trends in public interest, cultural perception, and conservation priorities [81,82]. The motivation for studying these species is often linked to visitor expectations and funding availability, as well as the animals’ perceived cognitive complexity and emotional appeal [81,83,84,85]. Research from cognitive and cultural psychology further suggests that mammals dominate not only public attention but also how people mentally categorize and recall animals. For example, Winkler-Rhoades et al. (2010) found that participants across urban and rural communities overwhelmingly named mammals when asked to list animals, with non-mammals mentioned less frequently unless culturally salient [86]. Moreover, the animals most readily named by participants often reflected symbolic or media-driven categories (e.g., “zoo animals” like lions or elephants), rather than local or ecologically relevant species [86]. This cognitive pattern mirrors institutional tendencies to prioritize species that align with public familiarity or narrative appeal.

This bias can lead to the underrepresentation of taxa whose welfare is equally important but less charismatic or symbolically prominent [84,87]. The persistence of these patterns may be shaped not only by logistical or institutional factors but also by differing welfare conceptions across the zoo field. Institutions vary in whether they prioritize biological functioning, the expression of natural behaviors, or the subjective experience of animals, differences which in turn may influence which species are studied and what technologies are used to assess them [24]. For example, birds and fish were underrepresented in the literature we examined, even though studies on these groups often encompassed multiple species, providing broader comparative insights [49,88,89]. This highlights an opportunity; expanding taxonomic representation does not necessarily require individual species studies but rather the inclusion of diverse taxa in group-level or multi-species designs.

### 5.4. Slow Progress in Expanding Taxonomic and Technological Scope

Even as new tools and frameworks emerge, a central concern remains: why have these innovations not led to more meaningful shifts in the diversity of taxa studied or technologies applied? Despite calls for increased taxonomic diversity in studies, little change has been observed in the past five years, with mammals continuing to dominate the research landscape [6,20]. This suggests that while technological advancements and increased research activity have expanded the field, they have not significantly shifted the focus away from traditional mammalian subjects. The limited use of technology beyond cameras further indicates a need for greater integration of multimodal monitoring approaches. Studies incorporating alternative monitoring tools—such as RFID systems for tracking nest usage in northern carmine bee-eaters, accelerometers for measuring activity in swans, and bioacoustics for assessing if captive golden mantella frogs recognize wild conspecific calls—have demonstrated their effectiveness in providing more nuanced perspectives [51,72,90]. These findings point to an opportunity for researchers to actively shift field norms by adopting taxonomically inclusive study designs and leveraging underutilized tools to ask richer, welfare-relevant questions across species.

### 5.5. Multimodal Technology in Research

Addressing these limitations requires exploring how combining technologies can overcome individual shortcomings and support more holistic welfare assessments. These examples highlight how combining technologies can overcome the limitations of any single tool, producing richer data and more robust welfare insights. Expanding multimodal designs to include a wider range of species—particularly those underrepresented in current research—offers a promising path toward more comprehensive welfare assessments. This also presents an opportunity to refine these systems for improved accessibility and lower implementation barriers across institutions. For instance, Hirskyj-Douglas and Kankaanpää (2022) developed a proximity-based interface that let white-faced sakis choose between audio and visual stimuli [23]. This system supported voluntary interaction and revealed individual preferences over time [23]. While designed for primates, its low-cost and modular setup shows how similar approaches could work across taxa [23]. As shown in Table 2, combining complementary tools like video monitoring with audio playback or RFID can help offset individual weaknesses and support more flexible and animal-centered welfare monitoring in line with ACI principles.

### 5.6. Study Limitations

Expanding the technological scope in this review introduced certain limitations. The inclusion of a diverse range of technologies, from traditional cameras to advanced AI systems, increased heterogeneity within the dataset, making direct comparisons between studies more complex. Additionally, many studies included multiple purposes, taxa, and technologies, which complicated efforts to assign studies to discrete categories. While this review quantified the presence of different technologies and taxa, more granular analysis, such as evaluating how specific technologies interact with particular taxa or research goals, would require qualitative coding or advanced categorization, which was outside the scope of this review at this time.

Furthermore, studies in which keywords related to zoos, aquariums, or technology did not appear in the title or abstract were likely missed during the search process. While this approach ensured that the studies included were directly relevant to the research questions, it may have excluded some relevant works where these terms were not explicitly stated. Future research could benefit from coding frameworks and broader search strategies that allow for more detailed cross-category analysis and inclusion of studies based on full-text screening.

This review relied primarily on PubMed for journal indexing, which may have limited the inclusion of studies from journals like *Applied Animal Behaviour Science*, where only a small fraction of total publications are indexed, potentially underrepresenting relevant research outside this database. For example, the 2019 study by Fuller, Heintz, and Allard on flipper-mounted time-depth recorders in penguins would have been an ideal inclusion in this review, as it exemplifies innovative, welfare-sensitive bio-logging in a zoo setting; however, it was not captured due to indexing limitations [105]. Additionally, this review limited its scope to five peer-reviewed journals selected based on prior reviews and relevance to zoo-based technology research; however, using journal selection as a pre-filter may have introduced publication bias or excluded relevant studies published in less conventional or newly established outlets. While these constraints affected the current analysis, they also point to methodological opportunities for future research, including the development of flexible coding systems and more inclusive search strategies that better capture the full range of unpublished or under-indexed work.

### 5.7. Future Directions

Building on these challenges, the final question becomes the following: how can future research shift current norms to support more inclusive, technology-integrated welfare strategies? Advancing zoo and aquarium research requires seizing the opportunity at the intersection of welfare, technology, and taxonomic diversity. Addressing the persistent gaps identified in this review will support more comprehensive and equitable welfare practices across species. Future research should focus on

1. Expanding Taxonomic Representation: More studies are needed on underrepresented taxa to ensure that welfare is comprehensive and inclusive of all species housed in zoos and aquariums.
2. Diversifying Technological Approaches Through ACI Principles: Integrating multiple monitoring technologies—such as bioacoustics, RFID, and infrared thermography—can offer a more holistic perspective on animal welfare beyond behavioral observations alone. To maximize their effectiveness, these technologies should also be grounded in animal–computer interaction (ACI) design principles that prioritize species-specific cognitive and sensory needs, enable voluntary interaction, and align with the ACI manifesto [1,19]. This includes developing more dynamic, adaptive enrichment systems using user-centered design frameworks tailored to the animals themselves.
3. Broadening Research Aims and Applications: In addition to identifying which technologies are used and which taxa are studied, future research should examine how these tools are applied to address different aspects of animal welfare. Current studies focus heavily on behavioral monitoring, often overlooking physiological, cognitive, and environmental indicators. Expanding the range of research aims to include areas such as thermal comfort, social dynamics, reproductive health, and species-specific enrichment can lead to more holistic welfare strategies. Aligning technology use with a wider range of care objectives will help ensure that scientific attention reflects the full spectrum of animal needs in managed care.

Overall, expanding the taxonomic, methodological, and ethical scope of zoo technology research is not only feasible but essential to ensure that all animals, not just the most visible or charismatic, benefit from scientific attention and innovation.

## 6. Conclusions

The results of this study underscore persistent and systemic biases in zoo and aquarium research, where mammals continue to dominate both taxonomic focus and technological use. While cameras remain the most frequently used tool, their prevalence reflects not just their utility but also an overreliance on what is convenient, familiar, or already installed, often at the expense of tools better suited to other species’ needs. Fundamental gaps remain in taxonomic representation, technological diversity, and research purpose [1,6,22]. These patterns suggest that research priorities may still be shaped more by legacy practices and human-centered preferences than by the full range of welfare needs in managed care. Greater attention must be paid to how technologies are applied to ensure that research meaningfully advances welfare across behavioral, physiological, and cognitive domains. Addressing these issues will require intentional, sustained efforts by researchers, institutions, and funders will have to reimagine how welfare is assessed and supported, ensuring that all species, not just the most visible or charismatic, benefit from scientific attention and technological innovation.

## Figures and Tables

**Table 1:** Proportion of papers meeting inclusion criteria by journal and year (2014–2024).

Year	Animals	JAAWS	JZAR	Zoo Biol.	AABS	Totals
2014	0.00%	0.00%	4.55%	2.17%	0.00%	6.72%
2015	0.00%	0.00%	3.85%	1.23%	0.00%	5.08%
2016	0.00%	0.00%	3.70%	9.20%	0.00%	12.90%
2017	0.00%	0.00%	14.81%	3.64%	0.00%	18.45%
2018	0.81%	0.00%	0.00%	13.85%	0.00%	14.66%
2019	0.33%	0.00%	3.45%	7.35%	0.00%	11.13%
2020	0.29%	1.30%	9.30%	1.59%	0.00%	12.47%
2021	0.25%	0.00%	5.41%	3.49%	0.00%	9.14%
2022	0.44%	0.00%	3.33%	5.10%	0.00%	8.87%
2023	0.26%	1.16%	7.41%	5.15%	0.00%	13.98%
2024	0.17%	2.41%	14.81%	5.71%	0.00%	23.11%
Totals	2.53%	4.87%	70.62%	58.49%	0.00%	N/A

Note. Percentages reflect the proportion of included studies (*n* = 125) by journal and year (2014–2024). JAAWS = *Journal of Applied Animal Welfare Science*; JZAR = *Journal of Zoo and Aquarium Research*; Zoo Biol. = *Zoo Biology*; AABS = *Applied Animal Behaviour Science*.

**Table 2:** Types of technology present in the study with strengths, weaknesses, and example studies from the review.

Technology Type	Strengths	Weaknesses	Example Studies
Cameras/Video Monitoring	Non-invasive for animals; widely accessible and applicable [25].	Reduced effectiveness depending on visibility; cannot assess internal physiological states [25].	[91,92,93]
Infrared Thermography	Non-invasive measurement of body temperature and stress-related thermoregulation [77].	Limited to surface temperature; individuals can have different temperature baselines [77].	[94,95,96]
Touchscreen Systems	Allows for cognitive enrichment and voluntary interaction [32].	Typically limited to primates; requires training and infrastructure [6,32].	[31,97,98]
Audio Playback/Recording	Captures vocalizations and supports cognitive engagement for auditory species [21].	Limitations in human understanding of animal communication [19].	[99,100,101]
Wearables/Biologgers/RFID	Tracks fine-scale movement, activity, social interactions; integrates with other sensors [26].	Requires animal habituation or training; invasive if poorly fitted [28].	[102,103,104]
AI/Deep Learning *	Reduces manual coding, captures subtle behaviors, and is scalable for long-term studies [12].	Primarily applied to mammals; requires large datasets for training [12].	[42,43,44,45,46,47]
Multimodal * (e.g., audio-equipped cameras, cameras + RFID, etc)	Combines behavioral, physiological, and environmental data for a broader welfare perspective.	May involve higher costs, complex data management, and reduced accessibility.	[52,53,54]

Terms with (*) are categories that were originally not categorized as part of the technologies defined for the study.

## Data Availability

The raw data presented in this study are available in Supplementary Material Table S1.

## Supplementary Materials

The following supporting information can be downloaded at: https://www.mdpi.com/article/10.3390/ani15121721/s1, Excel file Table S1: Raw data of the final publications selected for the systematic review.
